# Regorafenib with or without a programmed cell death protein 1 antibody as third‐line treatment for microsatellite stable metastatic colorectal cancer

**DOI:** 10.1002/cam4.5417

**Published:** 2022-11-13

**Authors:** Wen‐Zhuo He, Lei Wang, Chen‐Xi Yin, Jia‐Hong Yi, Ya‐Nan Jin, Chang Jiang, Gui‐Fang Guo, Liang‐Ping Xia

**Affiliations:** ^1^ VIP Region, Collaborative Innovation Center for Cancer Medicine Sun Yat‐sen University Cancer Center, State Key Laboratory of Oncology in South China Guangzhou P. R. China; ^2^ Department of Radiation Oncology The Third Affiliated Hospital of Kunming Medical University/Yunnan Cancer Hospital Kunming P. R. China; ^3^ Department of intensive care unit, Collaborative Innovation Center for Cancer Medicine Sun Yat‐sen University Cancer Center, State Key Laboratory of Oncology in South China Guangzhou P. R. China

**Keywords:** colorectal cancer, microsatellite stable, programmed cell death protein 1, regorafenib

## Abstract

**Background:**

Although the use of regorafenib plus nivolumab demonstrates promising outcomes in patients with refractory microsatellite stable (MSS) metastatic colorectal cancer (mCRC), this effect has not been substantiated in other studies. Moreover, a comparison between the outcomes of regorafenib and programmed cell death protein 1 (PD‐1) antibody combination therapy and regorafenib monotherapy remains unexplored. In this study, we aimed to assess whether regorafenib and PD‐1 antibody combination therapy is superior to regorafenib monotherapy as a third‐line treatment for MSS mCRC.

**Methods:**

Patients with MSS mCRC who received regorafenib and PD‐1 antibody or regorafenib monotherapy as third‐line treatment were eligible for inclusion.

**Results:**

In total, 179 patients were enrolled, of which 84 were administered regorafenib combined with a PD‐1 antibody and 95 were administered regorafenib monotherapy. Patients administered regorafenib combined with a PD‐1 antibody had similar progression‐free survival (PFS) as those on regorafenib monotherapy (median PFS was 2.4 months and 1.9 months, respectively, *p* = 0.086). The administration of regorafenib combined with a PD‐1 antibody resulted in significantly longer PFS than that seen with regorafenib monotherapy in both male (5.2 months vs. 2.4 months, *p* = 0.001) and female (3.9 months vs. 1.8 months, *p* = 0.037) patients without liver metastasis. Female patients with liver metastasis who were administered regorafenib combined with a PD‐1 antibody had shorter PFS than those administered regorafenib monotherapy (1.8 months vs. 2.0 months, *p* = 0.030).

**Conclusion:**

Liver metastasis and sex are predictors of survival benefit following the addition of a PD‐1 antibody to regorafenib in patients with MSS mCRC.

## Graphical abstract

Our study suggested that an age less than 60 years, in males, without liver metastasis was associated with benefit from treatment with the regorafenib and PD‐1 antibody combination as third‐line treatment for microsatellite stable metastatic colorectal cancer.

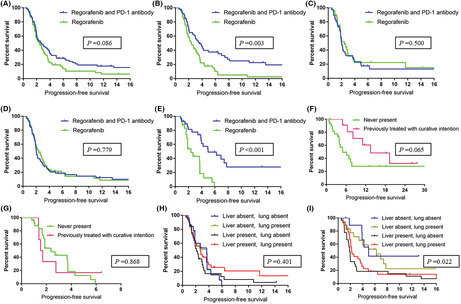

## 1 INTRODUCTION

Colorectal cancer (CRC) is a major health concern worldwide. Despite recent advances in treatment efficacy, therapeutic options for patients with refractory metastatic CRC (mCRC) remain limited.^1^ Although the use of programmed cell death protein 1 (PD‐1) antibodies in patients with microsatellite instability‐high (MSI‐H) mCRC is associated with improved outcomes, this clinical benefit has only been observed in a small proportion of mCRC cases.^2^ Moreover, more than 90% of mCRC cases are microsatellite stable (MSS). MSS mCRC has lower tumor mutational burden (TMB) and fewer neoantigens than those seen with MSI‐H mCRC. Thus, there are fewer tumor‐infiltrating lymphocytes in MSS mCRC, and therapy with PD‐1 antibodies has little efficacy in these patients.^3^ Hence, novel therapeutic strategies are urgently required for patients with MSS mCRC.

Regorafenib has been established as a standard third‐line treatment option for MSS mCRC.^4^ Recently, the combination of regorafenib and a PD‐1 antibody as a third‐line therapy was evaluated in several studies. In the REGONIVO study, wherein 24 patients with MSS mCRC were included, a partial response was observed in 33.3% of patients, whereas the median progression‐free survival (PFS) was 7.9 months.^5^ However, such effect was not observed in other studies. Wang et al. evaluated 18 patients and observed no partial response; moreover, 13 patients (69%) had progressive disease, and the median PFS was only 2 months.^6^ In another study, a combination of regorafenib and toripalimab yielded a response of 15.2% (5/33) and a median PFS of 2.1 months.^7^ Yang et al. conducted a study that involved 14 Chinese medical centers and reported a partial response of 5% (4/84 patients) and a median PFS of 3.1 months.^8^

These discrepant observations suggest that only a subset of patients may benefit from combination therapy. Indeed, several clinicopathological features are associated with the efficacy of PD‐1 antibodies. For example, sex can potentially affect the response of the immune system to antigens and thus the efficacy of the antibody.^9^ In several studies, male patients were reported to have a higher benefit from PD‐1 antibodies than female patients with melanoma and non‐small‐cell lung cancer (NSCLC).^10^, ^11^ Liver metastasis is a prognostic factor in patients receiving PD‐1 antibody treatment.^12^ Mechanistically, liver metastasis may impair the efficacy of PD‐1 antibodies by activating regulatory T cells and eliminating CD8^+^ T cells.^13^, ^14^ Recently, mCRC with homologous recombination deficiency (HRD) was reported to be associated with a higher tumor mutational burden (TMB), enriched immune cell infiltration, and sensitivity to immune therapy.^15^ The different distributions of these clinicopathological features may contribute to the discrepant efficacy outcomes of administering regorafenib with PD‐1 antibodies in various studies. In addition, these studies had a single‐arm design, and a comparison between regorafenib combined with PD‐1 antibodies and regorafenib monotherapy has rarely been reported.

To address this knowledge gap, we evaluated 179 patients with MSS mCRC. We compared the efficacy of regorafenib combined with PD‐1 antibodies to regorafenib monotherapy as third‐line therapy and explored the factors associated with clinical benefits from addition of a PD‐1 antibody.

## 2 MATERIALS AND METHODS

### 2.1 Patient selection

Records of patients who were diagnosed with mCRC, based on pathological samples, at Sun Yat‐sen University Cancer Center between January 2016 and December 2020 were retrospectively reviewed. Patients that met the following criteria were included^1^: diagnosed with MSS mCRC^2^; progressed to two lines of chemotherapy^3^; received regorafenib or regorafenib combined with a PD‐1 antibody as third‐line treatment. The study was approved by the Institutional Review Board of Sun Yat‐sen University Cancer Center (GZKJ2020‐02) and was performed in accordance with the Declaration of Helsinki. Written informed consent was obtained from the patients.

The dosage of regorafenib ranged from 80 to 160 mg [po, qd (D1–D21), q4w]. For patients administered regorafenib with a PD‐1 antibody, the dose of PD‐1 antibody was 200 mg (iv, 100 ml over 1 h ± 5 min, D1 and D15, q4w).

### 2.2 Clinical and gene sequencing data

Clinical information was retrospectively obtained from medical records. Routine blood analyses were performed using the Sysmex XE‐5000 Automated Hematology System (Shanghai, China). Next‐generation sequencing analyses were conducted in the Department of Molecular Diagnostics at our institute. TMB was determined by counting all nonsynonymous missense mutations that had not been previously reported as germline alterations. The detailed methods for next‐generation sequencing and TMB analyses are described earlier^16^ and are available as Data S1. Responses were evaluated according to the Response Evaluation Criteria in Solid Tumors, version 1.1.^17^ PFS was defined as the time from the first dose of treatment to progressive disease, death due to any cause, or the last follow‐up. The last follow‐up date was February 28, 2022. The median follow‐up time was 14.2 months.

### 2.3 HRD

HRD was defined in accordance with a previous report.^15^ Briefly, mCRC was defined as HRD if the tumor had any pathogenic or presumed pathogenic mutation in the following genes: *ABL1*, *ATM*, *ATR*, *BAP1*, *BARD1, BLM*, *BRCA1*, *BRCA2*, *BRIP1*, *CDK12*, *CHEK1*, *CHEK2*, *DNMT3A*, *ERCC1*, *ERCC4*, *FANCA*, *FANCC*, *FANCD2*, *FANCE*, *FANCF*, *FANCG*, *FANCL*, *MRE11*, *NBN*, *NONO*, *PALB2*, *RAD50*, *RAD51*, *RAD51B*, *RECQL4*, *RMI2*, *SFPQ*, or *WRN*.

### 2.4 Statistical analyses

All statistical analyses were performed using the SPSS version 13.0 software (Statistical Product and Service Solutions, Chicago City, Illinois State, United States). Frequencies and descriptive statistics were used to report patient characteristics and the chi‐square test was used for comparisons. The values of TMB and lymphocyte count did not conform to normal distribution. TMBs and lymphocyte counts were compared using the Wilcoxon rank‐sum test. Survival curves were calculated using the Kaplan–Meier method and compared using the log‐rank test. Statistical tests were two‐tailed, and differences were considered statistically significant at *p* < 0.05.

## 3 RESULTS

**FIGURE 1:**
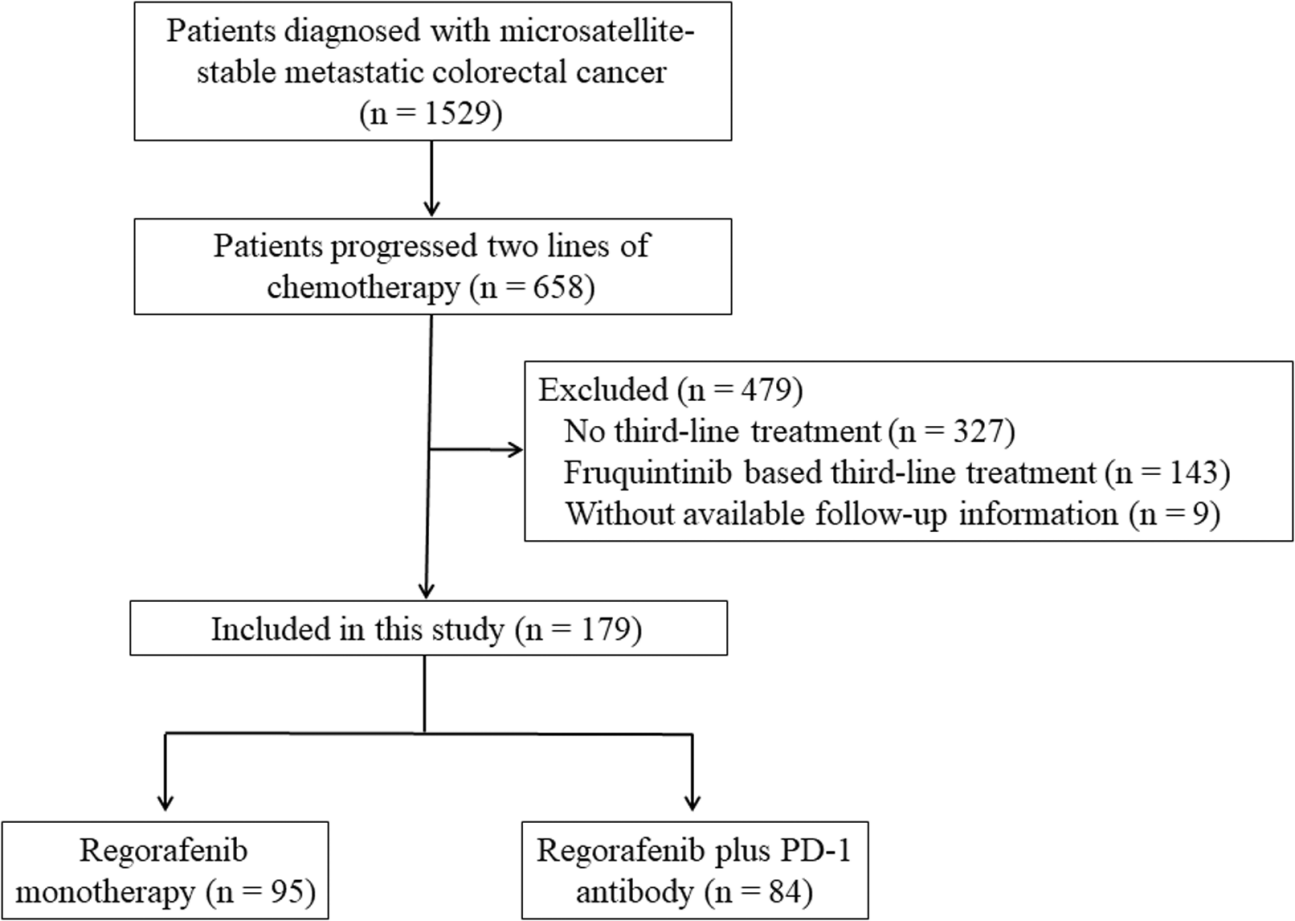
Flowchart depicting the patient selection process for the study

In total, 179 patients were included in the study **(**Figure 1 **)**, of which 106 were men (59.2%) and 73 (40.8%) were women. The median patient age was 52 years [interquartile range (IQR), 42–59 years]. A total of 55 (30.7%), 72 (40.2%), and 52 (29.1%) patients were diagnosed with right‐sided colon, left‐sided colon, and rectal cancers, respectively. A total of 125 patients (69.8%) had liver metastasis. Lung and peritoneal metastases were observed in 105 patients (58.7%) and 67 patients (37.4%), respectively.

**FIGURE 2:**
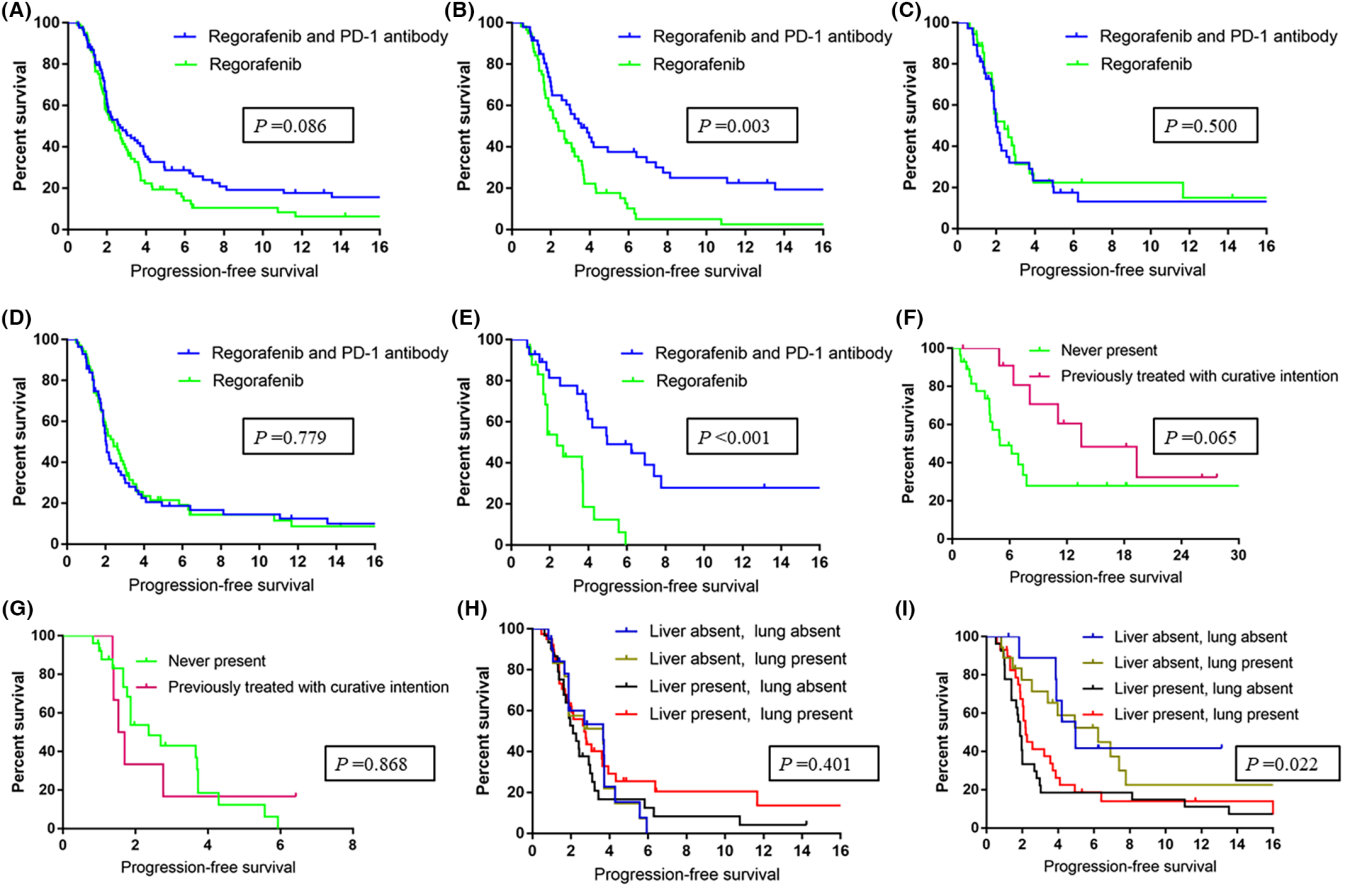
Progression‐free survival associated with regorafenib monotherapy and regorafenib combined with a PD‐1 antibody in all enrolled patients (A), male patients (B), female patients (C), patients with liver metastasis (D), and in patients without liver metastasis (E). Progression‐free survival associated with regorafenib combined with a PD‐1 antibody (F) and regorafenib monotherapy (G) in patients without a history of liver metastasis and in patients with liver involvement history but without active liver metastasis at the time of treatment. Progression‐free survival associated with regorafenib monotherapy (H) and regorafenib combined with a PD‐1 antibody (I) grouped by liver and lung metastasis

**FIGURE 3:**
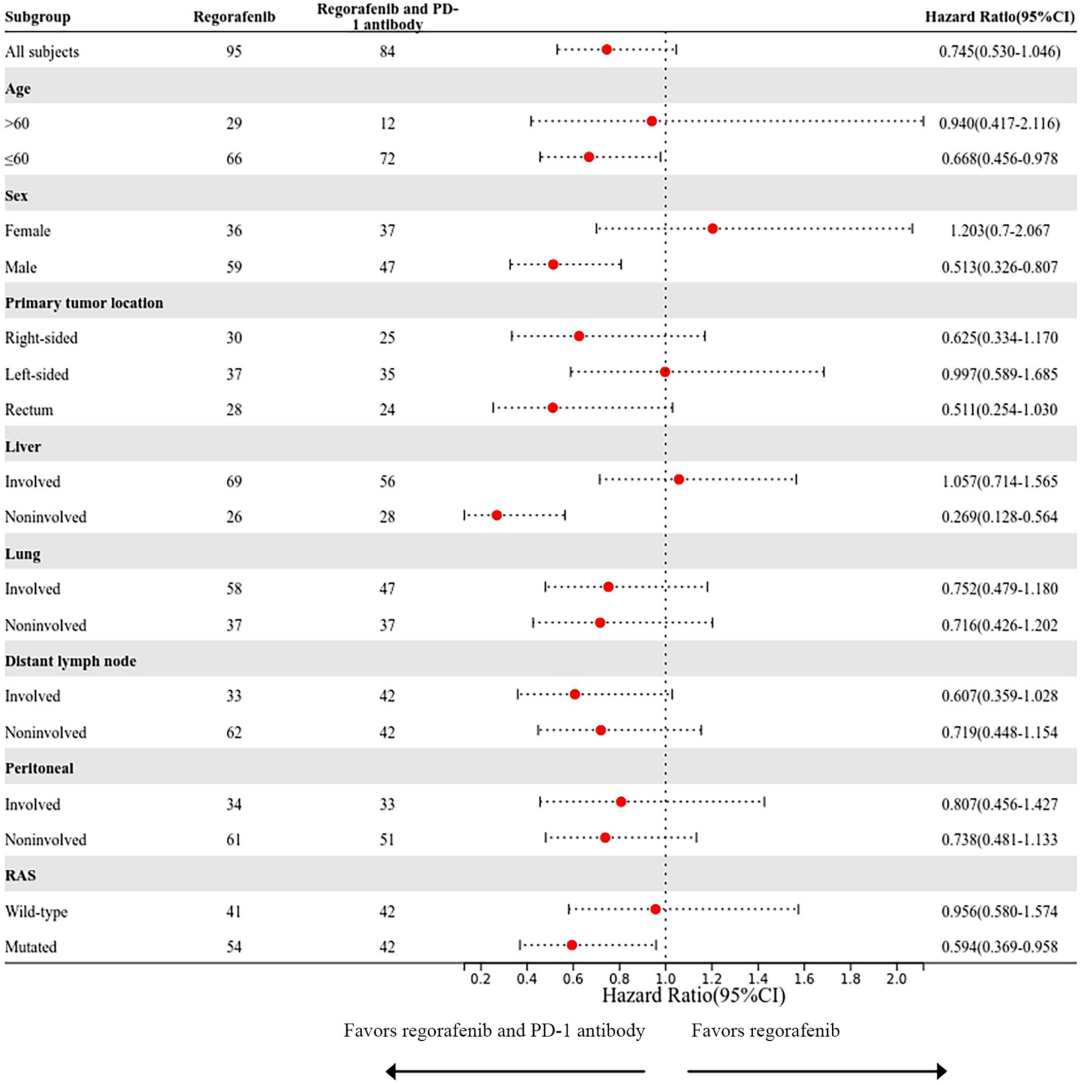
Forest plot of progression‐free survival according to patients' characteristics

Furthermore, 95 patients were administered regorafenib monotherapy and 84 were administered regorafenib with a PD‐1 antibody. For these 84 patients, the administered PD‐1 antibodies included nivolumab (35 patients), toripalimab (35 patients), sintilimab (11 patients), and camrelizumab (3 patients). Patients who were administered regorafenib combined with a PD‐1 antibody were younger and had less distant lymph node metastasis. Other clinicopathological features were similar between the two groups (Table 1). In the regorafenib combined with PD‐1 antibody group, 7 (8.3%) patients had partial responses and 30 (35.7%) patients had stable disease. No partial response was observed in the regorafenib monotherapy group, with stable disease observed in 37 (38.9%) patients. The disease control rate (DCR) was similar between the regorafenib with PD‐1 antibodies and regorafenib monotherapy groups (44% vs. 38.9%, *p* = 0.566). Among the enrolled patients, those who were administered regorafenib with PD‐1 antibodies had similar PFS as those who received regorafenib monotherapy (median PFS = 2.4 months and 1.9 months, respectively), as shown in Figure 2A (*p* = 0.086). The subgroup analysis suggested that an age less than 60 years in males, without liver metastasis and *RAS* mutation, was associated with benefit from treatment with the regorafenib and PD‐1 antibody combination (Figure 3).

**TABLE 1:** Patient and disease characteristics

Characteristic	Regorafenib, n (%)	Regorafenib and PD‐1 antibody, n (%)	*p*
Age			0.012
>60	29 (30.5)	12 (14.3)	
≤60	66 (69.5)	72 (85.7)	
Sex			0.448
Female	36 (37.9)	37 (44.0)	
Male	59 (62.1)	47 (56.0)	
Primary tumor location			0.931
Right‐sided	30 (31.6)	25 (29.8)	
Left‐sided	37 (38.9)	35 (41.7)	
Rectum	28 (29.5)	24 (28.6)	
Metastasis site			
Liver			0.417
Involved	69 (72.6)	56 (66.7)	
Noninvolved	26 (27.4)	28 (33.3)	
Lung			0.544
Involved	58 (61.1)	47 (56.0)	
Noninvolved	37 (38.9)	37 (44.0)	
Distant lymph node			0.049
Involved	33 (34.7)	42 (50.0)	
Noninvolved	62 (65.3)	42 (50.0)	
Peritoneal			0.646
Involved	34 (35.8)	33 (39.3)	
Noninvolved	61 (64.2)	51 (60.7)	
*RAS*			0.372
Wild‐type	41 (43.2)	42 (50.0)	
Mutated	54 (56.8)	42 (50.0)	
*BRAF*			0.050
Wild‐type	92 (96.8)	74 (88.1)	
V600E mutated	1 (1.1)	7 (8.3)	
Non‐ V600E mutated	2 (2.1)	3 (3.6)	

In the regorafenib alone group, the most common adverse event was hand‐foot syndrome (40.0%), followed by hypertension (33.7%) and proteinuria (9.5%). The most frequent adverse events in the regorafenib with PD‐1 antibody group were hand‐foot syndrome (33.3%), rash (13.1%), and hepatotoxicity (11.9%). We observed no grade 3 or 4 immunotherapy‐related toxicities.

Next, we explored the efficacy of the treatments based on sex. In men, the median PFS values were 3.4 months and 2.0 months for patients treated with regorafenib with PD‐1 antibodies and regorafenib monotherapy, respectively (*p* = 0.003, Figure 2B). In women, patients who were administered regorafenib combined with PD‐1 antibodies and those who were administered regorafenib monotherapy showed similar PFS (2.0 months vs. 1.9 months, respectively, *p* = 0.500, Figure 2C).

In patients with liver metastasis, the median PFS was 2.0 months in both groups (*p* = 0.779, Figure 2D). In patients without liver metastasis, those who were administered regorafenib combined with PD‐1 antibodies had significantly longer PFS than those in the regorafenib monotherapy group (median PFS = 4.2 months and 1.9 months, respectively), as shown in Figure 2E (*p* < 0.001). Among the 84 patients who were administered regorafenib with PD‐1 antibodies, 28 had no history of liver metastasis, whereas 12 had a history of liver involvement but without active liver metastasis at the time of treatment. These two groups of patients had similar PFS outcomes (4.2 months vs. 11.37 months, *p* = 0.065, Figure 2F). Among the 95 patients who were administered regorafenib monotherapy, 26 had no history of liver metastasis, whereas 6 had a history of liver involvement but without active liver metastasis at the time of treatment. These two groups showed similar PFS (1.9 months vs. 1.6 months, *p* = 0.868, Figure 2G).

In patients administered regorafenib monotherapy, no significant association was observed between PFS and the presence of liver or lung metastasis (*p* = 0.401, Figure 2H). In patients administered regorafenib with PD‐1 antibodies, liver metastasis was associated with shorter PFS regardless of the presence of lung metastasis (*p* = 0.022, Figure 2I).

**FIGURE 4:**
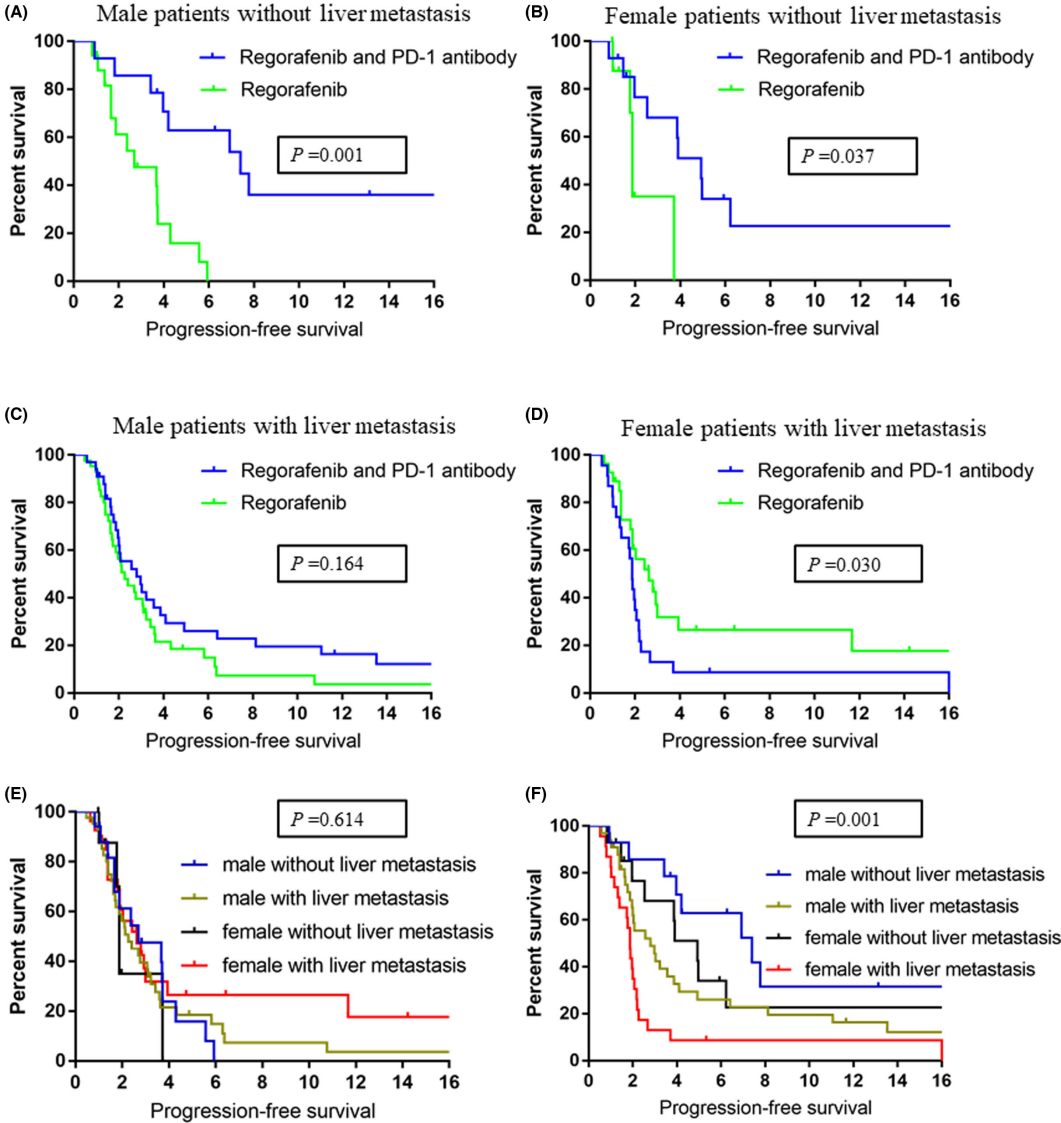
Progression‐free survival associated with regorafenib monotherapy and regorafenib combined with a PD‐1 antibody in male patients without liver metastasis (A), female patients without liver metastasis (B), male patients with liver metastasis (C), and female patients with liver metastasis (D). Progression‐free survival associated with regorafenib monotherapy (E) and regorafenib combined with a PD‐1 antibody (F) grouped by sex and liver metastasis

Given that being female and liver metastasis were associated with impaired efficacy of PD‐1 antibodies, we divided the patients into four groups (Table 2). We observed the highest partial response (4/31, 12.9%) among male patients without liver metastasis. In addition, 3/75 (4%) male patients with liver metastasis showed a partial response. By contrast, no partial response was observed in female patients. Regorafenib with PD‐1 antibodies led to longer PFS than regorafenib monotherapy in both male (5.2 months vs. 2.4 months, *P* = 0.001, Figure 4A) and female (3.9 months vs. 1.8 months, *P* = 0.037, Figure 4B) patients without liver metastasis. The regorafenib with PD‐1 antibody group had a similar PFS as did the regorafenib monotherapy (2.6 months vs. 2.0 months, *P* = 0.164, Figure 4C) group in the case of male patients with liver metastasis. Unexpectedly, female patients with liver metastasis who were administered regorafenib with a PD‐1 antibody had shorter PFS than those who were administered regorafenib monotherapy (1.8 months vs. 2.0 months, *P* = 0.030, Figure 4D).

**TABLE 2:** Comparison of patients' characteristics between patients with or without liver metastasis

Characteristic	Male		*p*	Female		*p*
	With liver metastasis, n (%)	Without liver metastasis, n (%)		With liver metastasis, n (%)	Without liver metastasis, n (%)	
Age			0.490			0.710
>60	21 (28.0)	11 (35.5)		7 (14.0)	2 (8.7)	
≤60	54 (72.0)	20 (64.5)		43 (86.0)	21 (91.3)	
Primary tumor location			0.748			0.022
Right‐sided	19 (25.3)	10 (32.3)		20 (40.0)	6 (26.1)	
Left‐sided	31 (41.3)	11 (35.5)		23 (46.0)	7 (30.4)	
Rectum	25 (33.3)	10 (32.3)		7 (14.0)	10 (43.5)	
Metastasis site						
Lung			0.134			0.449
Involved	41 (54.7)	22 (71.0)		27 (54.0)	15 (65.2)	
Noninvolved	34 (45.3)	9 (29.0)		23 (46.0)	8 (34.8)	
Distant lymph node			0.659			0.214
Involved	29 (38.7)	10 (32.3)		22 (44.0)	14 (60.9)	
Noninvolved	46 (61.3)	21 (67.7)		28 (56.0)	9 (39.1)	
Peritoneal			<0.001			0.204
Involved	16 (21.3)	19 (61.3)		19 (38.0)	13 (56.5)	
Noninvolved	59 (78.7)	12 (38.7)		31 (62.0)	10 (43.5)	
*RAS*			0.286			0.447
Wild‐type	39 (52.0)	12 (38.7)		20 (40.0)	12 (52.2)	
Mutated	36 (48.0)	19 (61.3)		30 (60.0)	11 (47.8)	
*BRAF*			0.625			0.213
Wild‐type	69 (92.0)	27 (87.1)		48 (96.0)	22 (95.7)	
V600E mutated	4 (5.3)	2 (6.5)		2 (4.0)	0 (0)	
Non‐ V600E mutated	2 (2.7)	2 (6.5)		0 (0)	1 (4.3)	
Homologous recombination deficiency			0.004			1.000
Yes	4 (9.8)	8 (44.4)		5 (17.2)	2 (15.4)	
No	37 (90.2)	10 (55.6)		24 (82.8)	11 (84.6)	
Response			0.056			0.065
Complete response	0 (0)	0 (0)		0 (0)	0 (0)	
Partial response	3 (4.0)	4 (12.9)		0 (0)	0 (0)	
Stable disease	26 (34.7)	15 (48.4)		14 (28.0)	12 (52.2)	
Progressive disease	46 (61.3)	12 (38.7)		36 (72.0)	11 (47.8)	

In patients administered regorafenib monotherapy, sex or liver metastasis was not associated with PFS (*p* = 0.614, Figure 4E). In patients administered regorafenib with PD‐1 antibodies, both female and liver metastasis were associated with shorter PFS (*p* = 0.001, Figure 4F).

**FIGURE 5:**
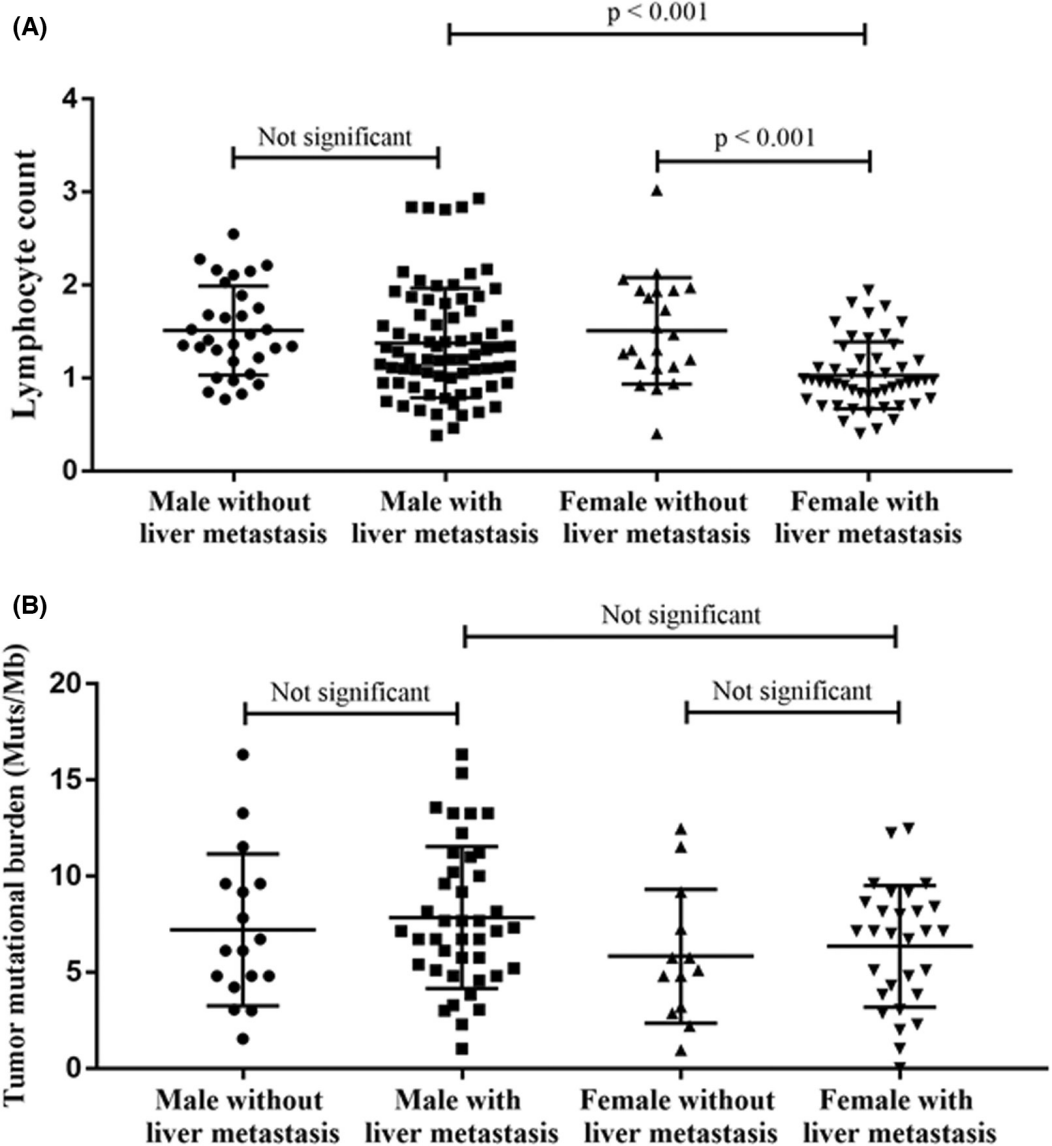
Comparison of peripheral lymphocyte count (A) and tumor mutational burden values (B) in patients grouped by sex and liver metastasis

Liver metastasis can impede the efficacy of PD‐1 antibodies through T‐cell elimination.^13^ In a previous study, it was observed that patients with NSCLC and liver metastasis had significantly lower absolute lymphocyte counts (ALC) than those without liver metastasis.^13^ We reviewed routine blood tests before the first dose of regorafenib or regorafenib combined with a PD‐1 antibody. Notably, we observed lower ALC in female patients with liver metastasis than that in patients without liver metastasis (median ALC was 0.96 vs. 1.39, *p* < 0001, Figure 5A). Male patients with liver metastasis had ALC that was similar to that in patients without liver metastasis (Figure 5A). mCRC with HRD has been reported to have higher levels of immune cell infiltration, and these patients might be potential candidates for immunotherapy. Herein, next‐generation sequencing data and TMB results were available for 101 patients. We observed the highest frequency of HRD alteration (8/18, 44.4%) in male patients without liver metastasis (Table 2). The other groups of patients had a relatively low HRD frequency, fluctuating between 9.8% and 17.2%. TMB was comparable between all the groups (Figure 5B).

## 4 DISCUSSION

Regorafenib is a multitarget inhibitor of the activity of angiogenic and stromal receptor tyrosine kinases, including vascular endothelial growth factor receptor, fibroblast growth factor receptor 1, and platelet‐derived growth factor receptor beta.^4^ The phase III CORRECT trial revealed a median PFS of 1.9 months, with a DCR of 41%, in 760 patients with refractory mCRC treated with regorafenib.^4^ PD‐1 plays a key role in restricting immune responses and facilitating self‐tolerance.^18^ The use of PD‐1 antibody has emerged as one of the most efficient strategies to boost antitumor immune responses. The combination of regorafenib and a PD‐1 antibody has attracted increasing interest following the promising response rate observed in the REGONIVO trial.^5^ However, several other studies evaluating the same treatment strategy failed to observe such a high response rate.^6^, ^7^, ^8^ These discrepant observations suggest that only a subset of patients may benefit from this combination. Our findings suggest that regorafenib combined with a PD‐1 antibody has similar efficacy as regorafenib monotherapy in unselected MSS mCRC as a third‐line treatment option. Furthermore, we show that the absence of liver metastasis and being male are associated with benefits of receiving the combination of regorafenib and a PD‐1 antibody. Treatment with regorafenib with a PD‐1 antibody, as compared with treatment with regorafenib alone, was associated with almost doubling of PFS among male patients without liver metastasis. By contrast, female patients with liver metastasis who were administered regorafenib with a PD‐1 antibody showed shorter PFS than those on regorafenib alone.

Not all metastatic sites of cancer have an equal impact on the efficacy of PD‐1 antibodies. The liver promotes immune tolerance in cases of viral infections and other diseases, and the tolerance mechanism can be hijacked by liver metastatic tumors.^19^ Indeed, various studies have suggested that liver metastasis can restrain the efficacy of PD‐1 antibodies. In patients with melanoma or NSCLC treated with pembrolizumab, the response rates were 56.3% and 30.6% in those without and with liver metastasis, respectively.^12^ Liver metastasis was also associated with significantly shorter PFS (5.1 months vs. 20.1 months).^12^ Furthermore, liver metastasis is associated with reduced tumor‐infiltrating CD8^+^ lymphocytes at the tumor infiltration front.^12^ A similar trend was observed in patients with NSCLC who were administered nivolumab.^20^ In the REGONIVO trial, patients with mCRC with liver metastasis also showed a reduced response rate.^5^ In a recent study comprising 95 patients with mCRC who were administered a PD‐1 antibody, a longer PFS was observed in those without liver metastasis than in those with the condition (4.0 months vs. 1.5 months, respectively).^21^ Our study results are consistent with previous observations. We also observed that patients with a history of liver involvement but without active liver metastasis at the time of combination treatment had similar PFS as did patients without a history of liver metastasis. This observation, together with the results of previous reports,^21^, ^22^ suggests that patients with incurable metastatic mCRC may benefit from aggressive local treatment targeting liver metastasis, as it can improve the patient's sensitivity to regorafenib combined with a PD‐1 antibody.

We further observed that the attenuating effect of the liver on the efficacy of regorafenib combined with a PD‐1 antibody was related to the sex of the patient. It is well known that sex can affect the process of both innate and adaptive immunity.^23^ Sex differences are associated with the prevalence and severity of various diseases, including autoimmune diseases, infectious diseases, and malignancies. Recently, the association between sex and immunotherapy efficacy in cancer was noted.^10^, ^24^ A systematic meta‐analysis including 20 randomized trials showed that the effect of immune checkpoint inhibitors is sex‐dependent and that male patients derive a greater therapeutic benefit.^11^ Herein, male patients without liver metastasis benefited the most from the combination of regorafenib and a PD‐1 antibody. By contrast, female patients with liver metastasis who were administered regorafenib with a PD‐1 antibody had reduced PFS compared with those who received regorafenib monotherapy. Furthermore, we observed that female patients with liver metastasis had the lowest ALC. Our observations support a previous preclinical finding that liver metastasis could induce apoptosis of antigen‐specific T cells, thereby promoting tumor immune escape.^13^ We also observed that male patients without liver metastasis had more frequent HRD alterations, which may explain their sensitivity to regorafenib combined with a PD‐1 antibody. The exact mechanism underlying the effect of sex on liver metastasis remains unclear, and further studies are needed to validate our observations. Our findings suggest that sex and liver metastasis should be considered as stratification factors in future clinical trials to avoid confounding factors.

We observed that an age less than 60 years was associated with survival benefit from treatment with the combination of regorafenib and anti‐PD1 antibody. Impaired antigen presentation and decreased number of lymphocytes infiltrating the tumor microenvironment has been reported for aged mouse model and patients.^25^ This may explain the limited efficacy of the PD‐1 antibody in patients older than 60 years. We also observed that *RAS* mutation was associated with survival benefit from the combination of regorafenib and anti‐PD1 antibody. Patients with CRC and *RAS* mutation are more likely to develop lung metastasis, which is a subset sensitive to immunotherapy.^26^ This may explain the survival benefit in patients with RAS mutation.

The major limitation of our study is that it involved a retrospective design. Second, 98 (54.7%) patients were alive and 24 patients entered clinical trials after disease progression. Therefore, we were unable to investigate and determine overall survival. Third, although 84 patients were administered regorafenib with a PD‐1 antibody, the actual drugs that were used varied. Fourth, next‐generation gene sequencing results were only available for 101 patients; therefore, we were unable to evaluate the HRD status in the remaining 78 patients.

Notwithstanding these limitations, we could compare the efficacy of regorafenib combined with PD‐1 antibodies and regorafenib monotherapy as a third‐line treatment option in a relatively large sample of patients with MSS mCRC. We demonstrate that male patients without liver metastasis benefited the most from the combination regimen. By contrast, female patients with liver metastasis were more likely to benefit from regorafenib monotherapy. These data suggest that liver metastasis and sex are predictive factors for the efficacy of PD‐1 antibodies in patients with MSS mCRC. Further prospective studies with a larger population are needed to validate our findings.

## AUTHOR CONTRIBUTIONS

**Wenzhuo He:** Investigation (equal); writing – original draft (equal). **Lei Wang:** Investigation (equal); resources (equal). **Chenxi Yin:** Investigation (equal); software (equal). **Jia‐hong Yi:** Formal analysis (equal); validation (equal). **Yanan Jin:** Methodology (equal). **chang jiang:** Software (equal). **guifang Guo:** Data curation (equal). **Liang‐Ping Xia:** Resources (equal); supervision (equal); writing – review and editing (equal).

## CONFLICT OF INTEREST

The authors have no relevant financial or non‐financial interests to disclose.

## ETHICAL APPROVAL STATEMENT

The study was approved by the Institutional Review Board of Sun Yat‐sen University Cancer Center (GZKJ2020‐02).

## PATIENT CONSENT STATEMENT

The study was performed in accordance with the Declaration of Helsinki. Written informed consent was obtained from the parents.

## Supporting information


Data S1

## Data Availability

The data in this study were deposited in the Research Data Deposit system of Sun Yat‐sen University Cancer (https://www.researchdata.org.cn/).
